# Complications, Not Minimally Invasive Surgical Technique, Are Associated with Increased Cost after Esophagectomy

**DOI:** 10.1155/2016/7690632

**Published:** 2016-12-08

**Authors:** Sue J. Fu, Vanessa P. Ho, Jennifer Ginsberg, Yaron Perry, Conor P. Delaney, Philip A. Linden, Christopher W. Towe

**Affiliations:** ^1^Division of Thoracic and Esophageal Surgery, University Hospitals Cleveland Medical Center and Case Western Reserve School of Medicine, Cleveland, OH, USA; ^2^Department of Surgery, University Hospitals Cleveland Medical Center and Case Western Reserve School of Medicine, Cleveland, OH, USA; ^3^Digestive Disease Institute, The Cleveland Clinic and Cleveland Clinic Lerner School of Medicine, Cleveland, OH, USA

## Abstract

*Background*. Minimally invasive esophagectomy (MIE) techniques offer similar oncological and surgical outcomes to open methods. The effects of MIE on hospital costs are not well documented.* Methods*. We reviewed the electronic records of patients who underwent esophagectomy at a single academic institution between January 2012 and December 2014. Esophagectomy techniques were grouped into open, hybrid, MIE, and transhiatal (THE) esophagectomy. Univariate and multivariate analyses were performed to assess the impact of surgery on total hospital cost after esophagectomy.* Results*. 80 patients were identified: 11 THE, 11 open, 41 hybrid, and 17 MIE. Median total cost of the hospitalization was $31,375 and was similar between surgical technique groups. MIE was associated with higher intraoperative costs, but not total hospital cost. Multivariable analysis revealed that the presence of a complication, increased age, American Society of Anesthesiologists class IV (ASA4), and preoperative coronary artery disease (CAD) were associated with significantly increased cost.* Conclusions*. Despite the association of MIE with higher operation costs, the total hospital cost was not different between surgical technique groups. Postoperative complications and severe preoperative comorbidities are significant drivers of hospital cost associated with esophagectomy. Surgeons should choose technique based on clinical factors, rather than cost implications.

## 1. Introduction

Minimally invasive techniques are a popular alternative to traditional open methods in nearly all surgical disciplines. An early concern with these techniques was potential increased operative cost relative to open surgery [1]. Studies of cost in abdominal surgical procedures, including cholecystectomy, appendectomy, reflux surgery, gastric bypass, ventral hernia repair, and colectomy, have refuted this concern and shown that minimally invasive techniques are associated with reduced ICU admissions, fewer complications, shorter length of stay, and decreased postdischarge resource utilization, all of which contribute to overall hospital costs [2–4]. As health care budgets come under scrutiny, the cost of surgical procedures should be assessed to ensure optimal use of health care resources relative to their clinical benefit. This is especially true for patients undergoing esophagectomy which is associated with high rates of complications and long term morbidity [5].

Advances in surgical techniques and perioperative standards have significantly decreased morbidity and mortality associated with esophagectomy [6]. A large body of research focused on clinical outcomes of minimally invasive esophagectomy (MIE) has demonstrated improved surgical and clinical outcomes, such as decreased blood loss, reduced length of stay, and fewer pulmonary complications [7–9]. Hybrid approaches, utilizing a combination of endoscopic and traditional approaches, have also been studied and show evidence of clinical benefit compared to traditional techniques [10, 11]. However, there is little data on economic outcomes of MIE and hybrid procedures compared to traditional approaches. The scant published data suggest MIE may result in decreased or similar costs [12]. A 2009 European study of MIE and open Ivor Lewis esophagectomy reported similar costs and safety, but this report was not adjusted for cost confounders in their analysis [13].

We aimed to analyze the costs associated with esophagectomy to assess whether a cost difference exists between minimally invasive, hybrid, and open approaches and, if so, which areas of the patient encounter contain cost differences. We hypothesized that there would be cost differences between minimally invasive and open techniques and that individual components, such as intraoperative cost or ICU cost, would account for the majority of the differences.

## 2. Methods

All patients undergoing esophagectomy at an academic medical center were identified via a prospectively maintained database between January 2012 and December 2014. Clinical data were extracted from the institution's database and were matched with institutional cost data pulled from UH-Socrates platform (Socrates Analytics, Cleveland, OH). This study was approved by the institution's Institutional Review Board. All data were analyzed using STATA/SE, version 13.0 (Stata Corp., College Station, TX).

Variables extracted from the clinical database included demographics, preoperative comorbidities, procedure characteristics, and postoperative complications. Preoperative characteristics extracted included age, body mass index (BMI), hypertension, coronary artery disease (CAD), prior chemotherapy or radiation, chronic obstructive pulmonary disease (COPD), diabetes, congestive heart failure (CHF), Zubrod class, and American Society of Anesthesiologists (ASA) class. Preoperative chemotherapy and/or radiotherapy was used as a proxy for tumor characteristics. Surgery type was classified as transhiatal (neck and abdominal incisions), open (open thoracotomy and open laparotomy), hybrid (either video assisted thoracic surgery (VATS) or laparoscopy for one part of the procedure), or purely minimally invasive (both VATS and laparoscopy). Both Ivor Lewis and tri-incisional esophagectomy could be performed using hybrid and MIE techniques. Full explanations of these procedures are available elsewhere [7]. The decision to use a specific technique was at the discretion of the attending surgeon. Reoperation and/or preoperative chemotherapy or radiation therapy were not contraindications to performing a minimally invasive approach. Complications were extracted from the database and were verified with chart review. Complications recorded included return to the operating room, postoperative infections, arrhythmias, respiratory issues (including reintubation and pneumonia), postoperative transfusions, and unexpected admission to the intensive care unit and were categorized as defined by the Society of Thoracic Surgeons [14]. Patients who had any recorded complication, regardless of complication severity, were categorized as having a complication.

The primary outcome of interest was total cost of the hospitalization. Actual hospital cost data were extracted from the institution's financial database. Patients with incomplete cost data were excluded. Costs were grouped into the following cost center categories: anesthesia, intensive care unit (ICU), laboratory, operating room, telemetry floor, and all other costs, which included cardiac, pharmacy, radiology, and physical therapy costs. The total cost for each patient was calculated by summation of the costs for all the cost center categories.

Descriptive analysis was performed to determine distribution of preoperative characteristics and complications. Due to nonnormal distribution of cost data due to several high outliers, nonparametric tests were utilized for cost analysis. Data are presented as median and interquartile range. Chi-square or Fisher's Exact test, as appropriate, was utilized to determine differences between groups for categorical variables. Kruskal-Wallis one-way analysis of variance was performed to determine whether there were cost differences between groups; pairwise comparisons between groups were then performed when differences were identified.* p* value less than 0.05 was considered significant.

Multivariable regression was utilized to examine the effect of preoperative and operative variables on cost, including pertinent preoperative variables as well as surgery type. Preoperative clinical variables, such as age, preoperative chemotherapy, and radiation, were included in the model as potential confounders. Complications were found to be a significant cost driver in the initial multivariate regression analysis and therefore we further stratified our multivariate regression analysis based on the occurrence of postoperative complications. Model 1 includes all patients. In order to differentiate the effect of complications on cost, we created two additional multivariable models. Model 2 includes only patients without complications and Model 3 includes only patients with complications.

## 3. Results

During the study period, 86 esophagectomies were performed by 4 surgeons. Eighty patients had complete medical records and cost data available and were included in the analysis (Table 1). Patient ages ranged from 32 to 85 years, with median age of 65 years. The most common preoperative comorbidity was hypertension (*n* = 44, 55%) followed by diabetes (*n* = 16, 20%). There were no differences in the preoperative characteristics between operative technique groups. The majority of the patients received preoperative chemotherapy (*n* = 52, 65%) and radiation therapy (*n* = 51, 63.8%), and the rate of preoperative chemotherapy and radiation was similar between operative groups. The hybrid approach was most common (*n* = 41, 51.25%) and was most commonly performed with a laparotomy and VATS (*n* = 38, 47.5%). The median length of stay (LOS) after esophagectomy was 8 (IQR 7–9) days. There was no difference in LOS between surgery types (*p* = 0.48). There were no deaths in the 30-day period following esophagectomy in this cohort. Complications occurred in 42 patients (52.5%). The most common complications were atrial fibrillation (*n* = 12, 15%), anastomotic leak (*n* = 11, 13.75%), and pneumonia (*n* = 11, 13.75%). Sixteen patients (20%) required return to the OR, which included patients that required endoscopy or bronchoscopy (even if no intervention was performed). A table which lists the frequency of all complications and reasons for return to OR is available in the online supplement (Table S1, in Supplementary Material available online at http://dx.doi.org/10.1155/2016/7690632). There was no difference in complication rate for the different procedure types (*p* = 0.88).

**Figure 1:**
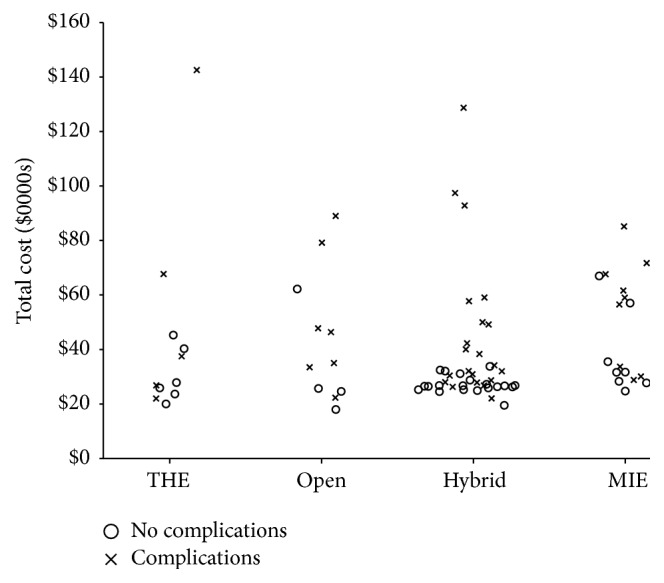
Range of cost for each procedure and by presence of postoperative complications. THE: transhiatal esophagectomy. MIE: minimally invasive esophagectomy.

The median total cost of the procedure and the associated hospitalization was $31,375 (IQR $26,487–$48,906).  Figure 1 depicts the range of total cost for each surgical technique. Operating room cost was the largest subtype of cost and accounted for $10,449 (IQR $9,108–$14,599) or approximately 33% of the total cost associated with the procedure. Median total cost and cost subgroups associated with each surgical technique are listed in Table 2. The total hospital costs for each procedure type were similar (*p* = 0.14). Anesthesia, OR, and non-ICU room and board floor costs, however, were different among the groups (*p* < 0.05). Specifically, MIE was associated with increased OR costs relative to each of the 3 other surgery types, increased anesthesia costs relative to transhiatal and hybrid esophagectomy, and increased non-ICU room and board costs relative to THE. There was a trend towards lower ICU costs in MIE patients (*p* = 0.08). The median operation time was 441 minutes for all surgeries. Operation time for MIE was significantly longer compared to the other surgeries (*p* < 0.01, Table 1). Operative time was related to OR cost (Figure S1), and this accounts for of the majority of the higher variable OR costs associated with MIE.

We also noted that, for all procedures performed, the occurrence of complications significantly increased total hospital cost. The unadjusted total cost increase for an associated postoperative complication was $17,804 (*p* = 0.0006, 95% CI $7,840–$27,758). The complication associated with largest cost increase was reintubation and was associated with a cost increase of $20,777. Anastomotic leak was associated with an average additional cost of $14,025.

By multivariable regression, ASA class IV, increasing age, preoperative coronary artery disease, and the presence of complications were associated with increased total hospital cost (Table 3). The total costs associated with each surgery type were statistically comparable. A preoperative diagnosis of hypertension appeared to be cost “saving” by $21,226. Among patients without complications, there was no single factor associated with increased (or decreased) total cost. Among patients with complications, age, preoperative coronary artery disease, and ASA class IV were associated with higher hospital cost, and hypertension was also cost “saving” in this group.

## 4. Discussion and Conclusions

In this study, we report that complications and preoperative characteristics, not surgical technique, affect inpatient hospital cost after esophagectomy. We found no difference in index hospitalization cost between the various surgical procedures performed. In the only other study that has assessed costs of minimally invasive and open esophagectomy, Parameswaran et al. found higher operative costs and lower inpatient care costs for MIE versus open transthoracic surgeries, but similar costs overall [13]. Notably, their cost analysis utilized calculated costs, whereas the current study was able to extract actual hospital costs. To our knowledge, the current study is the first to analyze the effect of esophagectomy surgery technique on hospital costs using actual patient costs. Our study corroborates the findings from Parameswaran and expands upon them using risk-adjusted analysis.

In breakdown of cost, we found that MIE was associated with increased OR, anesthesia, and telemetry floor costs among all patients and especially among patients who did not experience complications. The increased telemetry floor costs findings for MIE reflect increased non-ICU based room and board cost due to decreased proportion of time in the ICU. Despite not reaching statistical significance, we believe that MIE achieves cost parity with the other procedures by offsetting higher intraoperative cost with lower cost of postoperative care, largely by reducing cost related to ICU utilization. This is similar to results proposed in other fields of surgery since 2003 [3].

Our data supports a growing body of literature that minimally invasive procedures may be associated with increased OR cost but are potentially overall cost saving when other variables are considered [4, 15, 16]. This study corroborates those findings in that we also found that MIE was associated with longer and more expensive operations but was not associated with increased total hospital cost. Although the majority of the cost increase in MIE was related to longer operative time, other causes of higher MIE operating costs may be related to use of specialized and/or disposable instruments.

Because operative time is such a significant factor in cost, we believe that continued implementation of minimally invasive techniques may lead to cost saving over time as surgeons become more facile with these approaches and operative time decreases accordingly. Nguyen et al. reported a reduction of 108 minutes in MIE operating times in a series of 104 minimally invasive cases, eventually leading to shorter operating times in comparison to open esophagectomy [17].

While surgery type was not associated with significant cost differences, postoperative complications were revealed to be a significant cost driver in our analysis. When corrected for preoperative comorbidities and surgical technique, a hospital stay associated with a postoperative complication (or complications) was associated with an overall cost increase of $17,835. This supports previous data that postoperative events are associated with increased costs [18]. However, it should be noted that, for the purposes of this study, we did not differentiate severity of complications. Complication severity grade has been found to significantly increase hospital costs [19]. The relationship of postoperative complications on hospital costs may be related to different types of complications or their severity. In our cohort, for example, postoperative atrial fibrillation did not alter cost, while pneumonia and anastomotic leak led to increased cost. These subgroups also differed in that pneumonia was associated with increased ICU and radiology related cost, while anastomotic leak increased cost related to non-ICU room and board (and not ICU or radiology).

Two unexpected findings from this study were the independent effects of hypertension and coronary artery disease on hospital costs, as revealed by multivariate analysis. A preoperative diagnosis of hypertension was associated with cost* savings* of $21,226 among all patients and savings of $33,324 among patients that experienced a postoperative complication. Coronary artery disease was associated with additional costs of $16,189 overall, and again this effect was even more pronounced in patients with complications at $22,838, even when correcting for the occurrence of postoperative cardiac complications. Our data does not explain how hypertension could be cost saving, but we hypothesize that a diagnosis of hypertension may be a surrogate for improved preoperative medical management. Perioperative treatment of patients with diagnosis of hypertension with beta-blockers or aspirin, for example, may affect perioperative outcomes (or their severity) and therefore cost [20, 21]. Further research into this association is required.

Our study design has several limitations. Surgical technique was at the surgeon's discretion, creating bias in how patients were selected for the procedures. We have attempted to mitigate this bias in our analysis, by adjusting available confounders, but the potential for the effect remains given the retrospective nature of this study. Furthermore, the analysis was not adjusted to cancer stage, which may be a confounder in cost related to surgery. We have attempted to minimize the effect of clinical stage as a confounder by including preoperative chemotherapy and radiation, which we believe is a surrogate for advanced stage disease, in our multivariable analysis. Another limitation is that these cost data may not be generalizable to other institutions. Our use of postoperative pathways to decrease length of stay and avoid unnecessary radiographic studies may not be representative of a broader population. Additionally, the size of our cohort, while being larger than those of other studies on cost analysis of minimally invasive surgical techniques, is relatively small. Further subdivision into 4 groups may make the groups too small to identify differences even if they exist (type II error). The last weakness is that the data presented examines only inpatient hospital cost from the index hospital admission and does not reflect the true economic impact on the health care “system,” including costs associated with readmission. We believe that our data is nonetheless important in examining this “narrow” segment of cost because this cost remains the largest cost associated with esophagectomy and should include cost associated with most complications.

As the cost of health care is increasingly scrutinized, we believe that surgeons should take an active role in cost containment. Emerging technology has the potential to increase cost without leading to significant clinical benefit. This study demonstrates that minimally invasive techniques are no more costly than open procedures at our institution. Given that there were no differences in cost amongst these groups, we advocate that surgeons should choose technique based on clinical factors, not cost. We also show that patients with certain preoperative characteristics and postoperative complications can have significantly increased hospital costs. We would not advocate in any way that this data be used to “ration” care to patients that might cost less. Instead, we believe that this data supports using evidence based guidelines to reduce complications; reduction of cost would be a welcome secondary effect of having fewer complications. Future research is warranted to determine whether care pathways in esophagectomy can reduce associated hospital cost at a multi-institution level. In light of the escalating cost of healthcare, we believe that protocols that consider cost optimization (in addition to other clinical factors) should be implemented and studied.

## Supplementary Material

This table lists the frequencies of all complications and reasons for reoperation. The rate of complications was similar between operative technique groups.

## Figures and Tables

**Table 1:** Overview of study population.

	Median (IQR) or *N* frequency (%)					*p* value
	All	THE	Open	Hybrid	MIE	
*Patient variables*						

*N* (%)	80 (100)	11 (13.75)	11 (13.75)	41 (51.25)	17 (21.25)	

Age	65.2 (59.6−75.3)	67.4 (60.4−76.2)	65.1 (59.7−75.6)	64.7 (56.8−75.4)	63.4 (59.5−73.8)	0.891

Gender						0.241
Male	59 (73.8)	10 (90.9)	6 (54.6)	29 (70.7)	14 (82.4)	
Female	21 (26.2)	1 (9.1)	5 (45.5)	12 (29.3)	3 (17.7)	

Preoperative comorbidities or treatments						
Hypertension	44 (55.0)	4 (36.4)	6 (54.6)	25 (61.0)	9 (52.9)	0.550
CAD	15 (18.8)	4 (36.4)	1 (9.1)	7 (17.1)	3 (17.7)	0.502
Prior CTS	11 (13.8)	1 (9.1)	2 (18.2)	6 (14.6)	2 (11.8)	1.000
Preop XRT	51 (63.8)	7 (63.6)	5 (45.5)	29 (70.7)	10 (58.8)	0.455
Preop Chemo	52 (65.0)	7 (63.6)	6 (54.6)	29 (70.7)	10 (58.8)	0.729
COPD	10 (12.5)	1 (9.1)	2 (18.2)	5 (12.2)	2 (11.8)	0.955
CHF	5 (6.3)	2 (18.2)	1 (9.1)	2 (4.9)	0 (0.0)	0.189
Diabetes	16 (20.0)	2 (18.2)	1 (9.1)	9 (22.0)	4 (23.5)	0.904

Zubrod class						0.851
Normal Activity	33 (41.3)	5 (45.5)	4 (36.4)	15 (36.6)	9 (52.9)	
Symptomatic	47 (58.8)	6 (54.6)	7 (63.6)	26 (63.4)	8 (47.1)	

ASA class						0.573
II	7 (8.8)	2 (18.2)	0 (0.0)	4 (9.8)	1 (5.9)	
III	69 (86.3)	9 (81.8)	11 (100.0)	33 (80.5)	16 (94.1)	
IV	4 (5.0)	0 (0.0)	0 (0.0)	4 (9.8)	0 (0.0)	

*Operative outcomes*						

OR time (min)	441 (399−511)	330 (307−348)	449 (397−486)	437 (410−480)	527 (461−581)	<0.001

LOS (days)	8 (7−9)	7 (7−12)	8 (7−13)	8 (7−9)	7 (6−9)	0.476

Postoperative complications	42 (52.5)	5 (45.5)	5 (45.5)	23 (56.1)	9 (52.9)	0.912

Data presented as *n* (%) for categorical variables or median (interquartile range) for continuous variables.

*p* values indicate Fisher's Exact test for categorical variables and Kruskal-Wallis one-way test analysis of variance for continuous variables.

IQR: interquartile range. THE: transhiatal esophagectomy. MIE: minimally invasive esophagectomy. Preop: preoperative. CAD: coronary artery disease. CTS: cardiothoracic surgery. XRT: radiotherapy. COPD = chronic obstructive pulmonary disease. CHF: congestive heart failure. ASA: American Society of Anesthesiologist. LOS: length of stay.

**Table 2:** Median cost (% of total cost) [IQR] by cost center.

Cost center	Median (%) [IQR]					*p* value
	All patients (*n* = 80)	THE (*n* = 11)	Open (*n* = 11)	Hybrid (*n* = 41)	MIE (*n* = 17)	
OR	$10,449 (33%) [$9,108−14,599]	$7,703 (28%) [$7,274−10,060]	$10,903 (31%) [$9,992−16,816]	$10,099 (35%) [$9,081−11,300]	$15,732 (44%) [$11,721−25,218]	<0.001
Non-ICU room and board	$8,294 (26%) [$6,791−10,995]	$5,556 (20%) [$5,261−6,945]	$8,334 (24%) [$6,660−10,933]	$8,294 (29%) [$6,945−9,675]	$10,845 (31%) [$8,294−13,556]	0.005
ICU	$4,414 (14%) [$3,111−8,632]	$8,117 (29%) [$4,139−16,207]	$8,277 (24%) [$4,541−12,948]	$4,316 (15%) [$2,270−6,811]	$4,139 (12%) [$2,270−8,277]	0.085
Anesthesia	$2,204 (7%) [$1,918−2,713]	$1,644 (6%) [$1,413−2,146]	$2,374 (7%) [$2,016−3,443]	$2,148 (7%) [$1,920−2,525]	$2,946 (8%) [$2,382−4,237]	<0.001
Lab	$1,807 (6%) [$1,274−3,027]	$1,610 (6%) [$1,294−5,872]	$2,238 (6%) [$1,782−4,054]	$1,692 (6%) [$1,167−2,499]	$1,817 (12%) [$1,570−3,118]	0.271
Other^*∗*^	$3,706 (12%) [$749−5,744]	$3,411 (12%) [$1,311−5,937]	$6,067 (17%) [$1,544−10,509]	$3,518 (12%) [$0−4,907]	$4,151 (12%) [$2,241−5,550]	0.659

Total	$31,375 [$26,487−48,484]	$27,835 [$23,626−45,267]	$35,002 [$24,589−62,223]	$28,710 [$26,469−34,186]	$35,508 [30,101−61,623]	0.135

^*∗*^Other costs comprise pharmacy, radiology, cardiac, and physical therapy costs.

*p* values indicate Kruskal-Wallis one-way test analysis of variance for continuous variables.

IQR: interquartile range. ICU: intensive care unit. OR: operating room. THE: transhiatal esophagectomy. MIE: minimally invasive esophagectomy.

**Table 3:** Multivariate regression analysis.

Variable	All cases		Cases without complications		Cases with complications	
	Coefficient	*p* value	Coefficient	*p* value	Coefficient	*p* value
Complications	$17,385	<0.001				

Age (y)	$695	0.001	$240	0.196	$804	0.025

HTN	−$21,226	0.000	−$1,438	0.770	−$33,324	0.001

CAD	$16,189	0.006	−$5,831	0.502	$22,838	0.008

Preop ChemoXRT	−$5,173	0.252	$6,317	0.186	−$10,660	0.119

COPD	−$7,118	0.341	$5,156	0.555	−$17,171	0.152

CHF	$2,995	0.770	−$4,522	0.745	$6,547	0.680

Diabetes	−$630	0.910	$9,901	0.142	−$216	0.980

Zubrod symptomatic	$90	0.984	$4,887	0.270	$2,197	0.777

ASA class						
II	1		1		1	
III	$13,691	0.076	$7,406	0.246	$28,676	0.053
IV	$47,180	<0.001	n/a		$60,294	0.003

Procedure						
THE	1		1		1	
Open	$8,332	0.308	$2,304	0.746	$13,865	0.319
Hybrid	−$2,581	0.698	−$6,988	0.259	$13,858	0.907
MIE	$9,127	0.216	$6,457	0.353	$1,375	0.909

Constant	−$17,170	0.275	$2,845	0.838	−$11,753	0.655

Observations	80		38		42	

*R*-squared	.5546		.4349		.6713	

THE: transhiatal esophagectomy. MIE: minimally invasive esophagectomy. HTN: hypertension. CAD: coronary artery disease. CTS: cardiothoracic surgery. XRT: radiotherapy. COPD: chronic obstructive pulmonary disease. CHF: congestive heart failure. ASA: American Society of Anesthesiologist.

## Abbreviations

ASA: American Society of Anesthesiologists
BMI: Body mass index
CAD: Coronary artery disease
CHF: Congestive heart failure
COPD: Chronic obstructive pulmonary disease
ICU: Intensive care unit
IQR: Interquartile range
LOS: Length of stay
MIE: Minimally invasive esophagectomy
OR: Operating room
VATS: Video assisted thoracic surgery.
