# Supplementary material for: Complications, Not Minimally Invasive Surgical Technique, Are Associated with Increased Cost after Esophagectomy
Source: Minim Invasive Surg. 2016 Dec 8;2016:7690632. doi: 10.1155/2016/7690632 (PMC5178372; doi:10.1155/2016/7690632)
Supplement: Supplementary file 1 — This table lists the frequencies of all complications and reasons for reoperation. The rate of complications was similar between operative technique groups. [file 7690632.f1.pdf]

Supplemental tables and figures

Table S1: Complication types and frequencies

**Post-operative Events (n=80)**	**N Frequency (%)**
30 day return to OR	16 (20%)
Reoperation for bleeding	1 (1%)
Reoperation for anastomotic leak	2 (3%)
Reoperation for chyle leak	2 (3%)
Reoperation for empyema	1 (1%)
Endoscopy for evaluation and/or treatment of leak	2 (3%)
Bronchoscopy for atelectasis	1 (1%)
Surgical management of recurrent laryngeal nerve paresis	1 (1%)
Endoscopic evaluation and/or treatment of delayed gastric empyting (pyloric stenosis)	3 (4%)
Other (splenectomy, jejunostomy tube complications)	3 (4%)
Atrial fibrillation	12 (15%)
Leak of any grade	11 (14%)
Pneumonia	11 (14%)
Readmission within 30 days	11 (14%)
Reintubation	8 (10%)
Unexpected ICU admission	5 (6%)
Postoperative transfusions	4 (5%)
Laryngeal nerve paresis	3 (4%)
Urinary tract infection	3 (4%)
Pulmonary embolism	2 (3%)
Atelectasis	2 (3%)
Gastric outlet obstruction	2 (3%)
Chyle leak	2 (3%)
Tracheostomy	1 (1%)
Surgical site infection	1 (1%)
Other (esophageal stricture, sepsis, urinary retention)	3 (4%)
Any complication	42 (53%)

Abbreviations: ICU = Intensive Care Unit. OR = operating room.

Figure S1: OR costs plotted over OR time

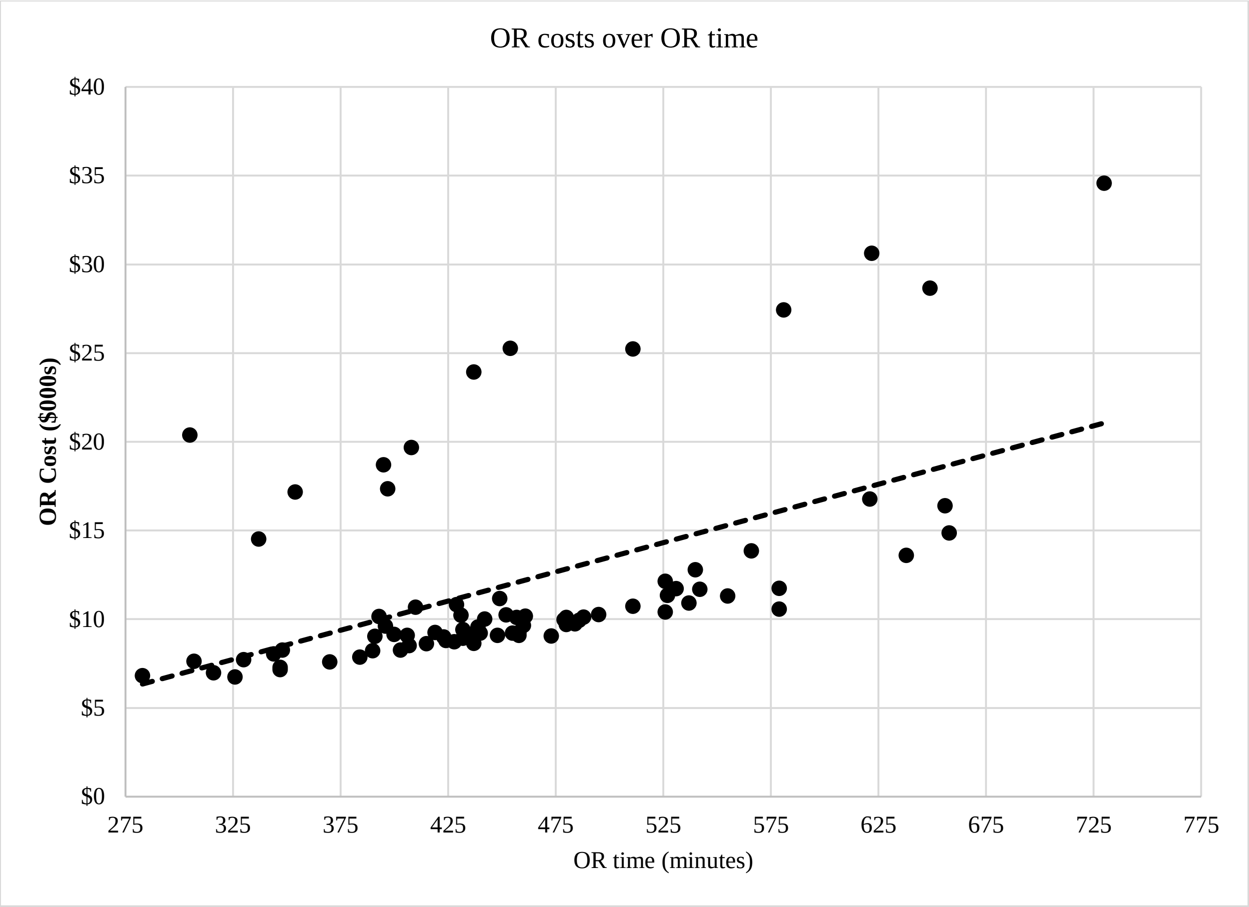

Abbreviations: OR = operating room.
